# *Helicobacter pylori* infection and inflammatory events: the extracellular vesicle-connect in driving gastrointestinal tract cancers

**DOI:** 10.3389/fmed.2024.1444242

**Published:** 2024-11-14

**Authors:** Prabin Bawali, Abhisek Brahma, Smruti Ranjan Rana, Aranya Pal, Asima Bhattacharyya

**Affiliations:** School of Biological Sciences, National Institute of Science Education and Research (NISER) Bhubaneswar, An OCC of Homi Bhabha National Institute, Khurda, Odisha, India

**Keywords:** extracellular vesicle (EV), gastric cancer, gastrointestinal tract, inflammation, microbiota, outer membrane vesicle (OMV), OMV fusion, reactive oxygen species

## Introduction

The association of chronic inflammation with tumorigenesis is well-established. The pathogenesis of *Helicobacter pylori*-mediated gastric cancer (GC) is an appropriate example of inflammation-driven tumorigenesis. This Gram-negative pathogen resides in the human gastric mucosa and incites a continued inflammatory response marked by immune cell infiltration, production of cytokines, chemokines and reactive oxygen species (ROS) (1). These events generally activate diverse signaling pathways in the infected gastric epithelial cells (GECs), which play crucial roles in carcinogenesis. This process is orchestrated by the interplay of *H. pylori*-derived outer membrane vesicles (OMVs) and host-cell-derived extracellular vesicles (hEVs), which serve as secret carriers, transport a diverse cargo of biomolecules, including the carcinogenic cytotoxin of *H. pylori*, the cytotoxin-associated gene A (CagA) (2, 3). Depending on the duration of infection and the host response, the cargoes of the vesicular packages vary. As the EV released by the infected GECs and *H. pylori*-derived vesicles can be carried to the distal parts of the body, we believe these vesicular bodies trigger signaling events in distal organs. In fact, evidence suggests that *H. pylori* is not only associated with gastric pathogenic events but also contributes to the disease pathogenesis of various other organs (2). However, the gastrointestinal tract (GIT) presents another layer of complexity due to the dynamic population of the GIT microbiota. These microorganisms may influence the prevalence of *H. pylori* and alter the immunopathogenic outcome (4). Despite some understanding, studies in this field are in their early stages. Here, we present our analysis and viewpoint on the potential involvement of *H. pylori* infection-generated EVs in fostering gastric adenocarcinoma as well as other gastrointestinal tract cancers.

## Immunopathogenesis of *H. pylori*-mediated GC

*H. pylori* infection is associated with approximately half of the global population and is a causative factor for GC. As an extracellular pathogen, *H. pylori* is mainly equipped to infect the human gastric epithelium. *H. pylori* virulence factors trigger the production of proinflammatory mediators in the infected GECs which drive the progression of gastritis, peptic ulcer, mucosa-associated lymphoid tissue (MALT) lymphoma and in some cases, GC. The “pathogenic” virulence factor CagA is considered a type I carcinogen for GC. The risk of GC significantly increases when CagA is accompanied by certain subtypes of other cytotoxins such as, the virulence factor vacuolating cytotoxin A (VacA) and the blood group antigen binding “colonizing” virulence factor/adhesin (BabA) (5). Polymorphism of inflammation-regulating host genes largely influences pathogenesis by *H. pylori*. Although, both CagA and VacA allow persistence of the pathogen by suppressing host immune responses, CagA can promote inflammation in the gastric epithelium by inducing the nuclear factor-κB (NF-κB) pathway and interleukin-8 (IL-8) secretion in some hosts. Both of these factors are found at very high level in the blood of GC patients. In addition, higher expression level of a T helper type-1 (T_H1_) cell cytokine IL-1β and decreased level of the anti-inflammatory cytokine IL-10 are associated with increased gastric atrophy and distal GC (6). *H. pylori* virulence factor urease B (UreB) also increases the expression of the proinflammatory cytokine tumor necrosis factor-α (TNF-α) and can promote carcinogenic events in genetically-susceptible hosts. Cyclooxygenases (COX1 and COX2) are the other inflammatory mediators released by *H. pylori*-infected GECs. In response to these inflammatory mediators, GECs and immune cells in the infected gastric epithelium upregulate ROS generation (1). Downregulation of ROS-scavengers ascorbic acid (7) and GRP78/BiP (8) also promotes gastric carcinogenesis. *H. pylori*-induced ROS generation (9) and the degradation of prolyl hydroxylase 3 (PHD3) (10) can stabilize the oncogenic transcription factor hypoxia-inducible factor 1α (HIF1α) causing transcriptional activation of many hypoxia-responsive oncogenes. ROS also initiate mutagenic events which promote GC progression. In addition, the suppression of apoptosis significantly aids in the establishment of *H. pylori* infection (11) and the survival of mutant cells. However, a T_H_1 cytokine, interferon-γ (IFN-γ) induces apoptosis of *H. pylori*-infected GECs (12). Therefore, the risk of GC development is heavily dependent on the host immune responses and signaling events.

## *H. pylori* infection and extragastric GIT cancers

*H. pylori* shows tropism to the gastric pit (13) but various extragastric manifestations of *H. pylori* infection have been reported in recent times (14). Owing to the fecal-oral transmission route, *H. pylori* has been isolated from several parts of the GIT and shown correlation with several GIT cancers. The presence of CagA-positive *H. pylori* in the oropharyngeal region can mount a local immune response against human papillomavirus (HPV) infection (15, 16) but their co-occurrence has also been linked to the advancement of oropharyngeal carcinoma (17). Emerging evidences link *H. pylori* to colorectal, pancreatic and hepatobiliary cancers as well (18). Gallbladder premalignant lesions are found to be correlated with gastric colonization of *H. pylori* and resultant ROS production (19). *H. pylori* promotes autoimmune pancreatitis by stimulating inflammatory responses in the pancreatic tissue leading to the increased risk of pancreatic cancer (20). However, so-far, only one case-study has reported about *H. pylori* bacteraemia (21) which is indicative of the systemic spread of the virulence factors instead of “true” bacterial colonization in extragastric tissues.

## *H. pylori* infection and the GIT microbiota

GIT oncogenesis is complicated due to the presence of trillions of microorganisms in the GIT. Other than *H. pylori*, which is the most predominant bacterium, the human stomach itself houses *Lactobacillus, Streptococcus, Prevotella, Hemophilus, Neisseria* and *Rothia*, to name a few. These microbes belong to the five most abundant phyla- *Proteobacteria, Firmicutes, Actinobacteria, Bacteroidetes* and *Fusobacteria* (22). The abundance of these microbes varies depending on *H. pylori* infection, dietary habits, race, age and disease states.

*H. pylori* not only rewires the α-diversity of the normal gastric microbiota but also of the entire gut microbiota that has a possible connection with various other GIT cancers (23). The gastric microbiota composition differs significantly between *H. pylori*-positive and *H. pylori*-negative patients. In the *H. pylori*-positive group, *Proteobacteria* are more prevalent and coexist with *H. pylori* through mutualistic relationships, taking advantage of the altered pH while *Firmicutes* might be supressed due to competition, modulated pH and microenvironment. *H. pylori* infection has been reported to decrease pathways involved in carbohydrate and lipid metabolism which significantly downregulates *Firmicutes* (24). On the contrary, in *H. pylori*-negative group, both *Proteobacteria* and *Firmicutes* are the predominant phyla. It is worth mentioning that the gastric microbiota diversity is decreased by *H. pylori* colonization (25). Studies have found correlations of other bacteria with *H. pylori*-mediated GC progression. For example, gastric neoplasia progresses much faster upon the introduction of intestinal pathogenic bacteria to solely *H. pylori*-infected germ-free insulin-gastrin mice (26). Robinson et al. (27) have reported that GC has diverse bacterial DNA content, with *Pseudomonas* sp. being dominant but not *H. pylori*. Once initiated by *H. pylori* infection, human GC progression, beyond the adenocarcinoma stage, may not depend on *H. pylori* since the gastric microbiota consists of primarily oral and intestinal bacteria as observed after *H. pylori* eradication (25). These reports point to the possibility that the GIT microbiota strongly dictates GC progression. Further studies are required to discern the exact mechanism.

## EVs ferry oncogenic as well as inflammatory factors and regulate *H. pylori*-mediated GC pathogenesis

Over the last two decades, we have become aware that gastric colonization of *H. pylori* can lead to systemic effects and can influence extragastric disease manifestations including extragastric oncogenesis (28, 29). One of the mechanisms proposed for the extragastric association involves the release of OMVs by *H. pylori* and infected cell-derived hEVs which deliver virulence factors and genetic material to host cells. These OMVs and hEVs exhibit intricate functions and act as conveyance systems for virulence factors, ROS-regulators, immune and inflammatory modulators. *H. pylori* OMV-packed gamma-glutamyl transpeptidase (GGT), another virulence factor, also induces ROS and promotes chronic inflammation in the GIT (2). Systemic low-grade inflammation is associated with atherosclerosis in *H. pylori*-infected males (30) and CagA influences the process (31). The main players behind these inflammatory events are IL-8 and NF-κB (32). EVs released by *H. pylori*-infected GECs carry CagA, become blood-borne and deliver the oncogenic factors to the distal parts of the body contributing to atherogenesis and systemic inflammation (33). Interestingly, a study by Olofsson et al. (34) has shown that CagA, VacA, BabA, urease and more than 300 other *H. pylori* proteins appear in the *H. pylori* infection-derived vesicles. These vesicles can engage in crosstalk by either competing for host cell binding sites or act synergistically to enhance their virulence or immunomodulatory properties.

## Fusion of *H. pylori* OMVs with other microbial OMVs and extragastric cancers, our perspective

Among the GI organs, the human stomach is unique since the pH in its lumen can be very low. A healthy human stomach has a pH ranging between 1.5–3. Interestingly, the long-term use of proton pump inhibitors (PPIs), which are widely used to prevent gastric ulcers, has been associated with an increased risk of GC (35). PPIs increase the stomach pH by inducing hypochlorhydria. Alkaline pH, due to either hypochlorhydria or chronic inflammation or microbial metabolites, helps in the growth of extragastric pathogens, mainly oral microbes such as *Streptococcus anginosus, Slackia exigua, Peptostreptococcus stomatis* in the stomach (36). However, the overall microbial heterogeneity in the stomach gets decreased which modulates GC development. pH neutralization by *H. pylori* can also cause the accumulation of pathogenic microbes in the stomach contributing to oncogenesis (37).

From the above discussion about the GIT microbiota influence on GC and other GIT cancer progression, we believe that OMVs released in an *H. pylori-*infected GIT are not simply bystanders. Interestingly, *H. pylori* OMVs can alter the hepatocyte-derived exosome contents, activate hepatic satellite cells and downregulate E-cadherin leading to hepatic fibrosis (38). *H. pylori*-mediated duodenal ulcer is associated with the OMV-encapsulated Omp30 release (39). Both of these diseases have definite premalignant potentials. Exosomal CagA promotes colitis by caudal type homeobox 2 (CDX2)-dependent claudin-2 upregulation in the intestinal epithelial cells (40). Claudin2 is long known for its assocaiation with colon cancer. Though not studied in *H. pylori* infection, glyceraldehyde-3-phosphate dehydrogenase (GAPDH) secreted by Gram-negative bacteria helps in OMV-membrane fusion and is implicated in spreading bacterial cytotoxins to remote cells (41). This fusogenic enzyme is employed by the Gram-negative bacteria *Moraxella xanthus* to ascertain topological fusion of its own OMVs with other Gram-negative bacterial OMVs (42). Although the mechanism of OMV release by Gram-positive bacteria is not definitively understood, under some circumstances they release OMVs (43). Therefore, the GIT OMV pool from both Gram-negative and Gram-positive bacterial population may function as a lush haul of oncogenic factors and immune modulators not only in the GIT but also in other organs, post their intestinal absorption. Likely, the influence of the collective OMVs and fused-OMVs in the GIT will decisively steer the rate and course of oncogenesis in the GIT as well as in other organs. As OMVs can invade host cells and even accumulate near tumor cells to influence the tumor microenvironment (32, 44), their synergistic effects on GIT cancer need to be thoroughly studied.

EV fusion with the target cell membrane has been observed under low pH condition although the fusion mechanism is not clearly known (45). In experimental conditions, low pH allows bacterial OMV fusion (46). The stomach is the likely organ in the human body which, in physiological conditions, might allow EV lipid-membrane fusion due to the low pH condition in the stomach lumen. The membrane-bending physical and electrical conditions are created at low pH which leads to the rearrangement of lipid polar groups (47). Although there is no experimental evidence yet, but by going through the existing literature, we believe that fused OMVs can be formed at the low pH of the human stomach.

**Figure 1:**
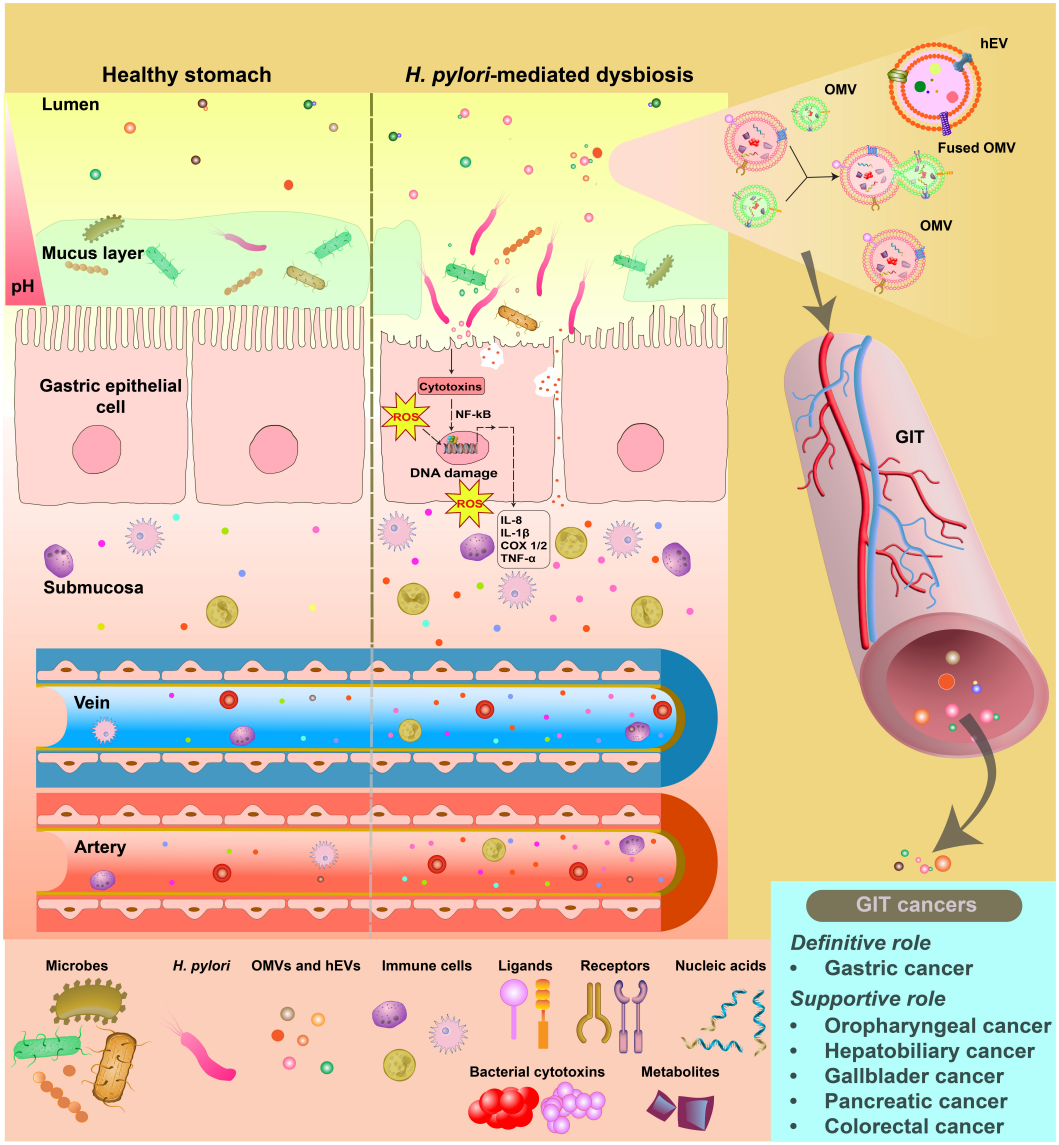
The GIT microbiota-derived OMVs help in strengthening the carcinogenic potential of *Helicobacter pylori* beyond the human stomach. *H. pylori* resides in the human stomach and incites persistent inflammatory reactions and oxidative stress. hEVs and OMVs released by *H. pylori* carry cytotoxins as well as other factors which contribute to GC pathogenesis. Low pH in the lumen of the human stomach opens up the possibility of *H. pylori*-derived OMVs to fuse with OMVs originating from other pathogenic bacteria in the stomach. In addition to bind with their own target cells, fused OMVs have possibilities to bind with the cell membrane of various off-target cells of the GIT and cause carcinogenesis. Spreading of fused OMVs through the blood vessels opens the possibility of distribution of *H. pylori* cytotoxins to other distal organs in the body as well. The figure is not drawn to the scale.

Figure 1 summarizes our viewpoint regarding the OMV-OMV fusion possibilities in the acidic environment of human stomach.

## Conclusion, lacunae and future perspectives

The potential of pH-dependent multi-microbial OMV fusion might lead to changes in the overall composition and properties of the resultant fused OMV, thereby broadening the receptivity of various off-target extragastric regions with unique pH environments (48). pH regulates the cellular uptake and release of EVs, inducing inflammation-mediated diseases such as the Alzheimer's disease, pulmonary diseases, dermatological disorders or cancer. Comprehending these complex relationships is crucial to interpret the role of *H. pylori* and other microbes in these diseases, demanding targeted research to facilitate precise therapeutic modalities. OMVs are considered as good vaccine candidates. The compositional attributes of *H. pylori* OMVs and the intricate local gastrointestinal *milieu* and microbiota introduce novel dimensions for OMV-OMV fusion. Nevertheless, the precise molecular determinants orchestrating their preferential fusion with cells of extragastric organs, particularly within the distinct and heterogenous microenvironment of these organs, have yet to be comprehensively defined. Future therapeutic strategies are poised to harness the transformative potential of these nanoscale vesicles loaded with therapeutic biomolecules. Designing customized bacterial OMVs with treatment agents, surface engineering such as membrane protein modification or ligand incorporation may assist in the drug delivery attempts at the desired site of action. While bioengineered hybrid EVs have been generated for diagnostics and drug delivery, OMV-OMV fusion in the body still remains an enigma. Their fusion opens up the possibility of binding the fused OMVs with other nonconventional host cells based on the compatible ligand-receptor interaction and portrays the possible reasons behind the association of *H. pylori* with extragastric cancers. Due its implication in the disease pathogenesis, the OMV-OMV fusion phenomenon warrants for in-depth research. Particularly, hybrid vesicles that integrate EVs of biological origin with natural cargo-carrying capacity and synthetic EVs with customizable features will augment the latter's compatibility, therapeutic potential and specificity (49). With the advancement of EV research and bioengineering, the potential of EVs in personalized, targeted and efficient therapies will become clearer while minimizing systemic toxicity.

## References

[1] Butcher LD den Hartog G Ernst PB Crowe SE. Oxidative stress resulting from *Helicobacter pylori* infection contributes to gastric carcinogenesis. Cell Mol Gastroenterol Hepatol. (2017) 3:316–22. 10.1016/j.jcmgh.2017.02.002 28462373 PMC5404027

[2] Wang C Li W Shao L Zhou A Zhao M Li P. Both extracellular vesicles from *Helicobacter pylori*-infected cells and *Helicobacter pylori* outer membrane vesicles are involved in gastric/extragastric diseases. Eur J Med Res. (2023) 28:484. 10.1186/s40001-023-01458-z 37932800 PMC10626716

[3] Yanez-Mo M Siljander PR Andreu Z Zavec AB Borras FE Buzas EI. Biological properties of extracellular vesicles and their physiological functions. J Extracell Vesicles. (2015) 4:27066. 10.3402/jev.v4.27066 25979354 PMC4433489

[4] Xu W Xu L Xu C. Relationship between *Helicobacter pylori* infection and gastrointestinal microecology. Front Cell Infect Microbiol. (2022) 12:938608. 10.3389/fcimb.2022.938608 36061875 PMC9433739

[5] Chang WL Yeh YC Sheu BS. The impacts of *H. pylori v*irulence factors on the development of gastroduodenal diseases. J Biomed Sci. (2018) 25:68. 10.1186/s12929-018-0466-9 30205817 PMC6131906

[6] Peek RM Jr. Blaser MJ. *Helicobacter pylori* and gastrointestinal tract adenocarcinomas. Nat Rev Cancer. (2002) 2:28–37. 10.1038/nrc703 11902583

[7] Drake IM Davies MJ Mapstone NP Dixon MF Schorah CJ White KL. Ascorbic acid may protect against human gastric cancer by scavenging mucosal oxygen radicals. Carcinogenesis. (1996) 17:559–62. 10.1093/carcin/17.3.559 8631145

[8] Dixit P Suratkal SS Kokate SB Chakraborty D Poirah I Samal S. Siah2-GRP78 interaction regulates ROS and provides a proliferative advantage to *Helicobacter pylori*-infected gastric epithelial cancer cells. Cell Mol Life Sci. (2022) 79:414. 10.1007/s00018-022-04437-5 35816252 PMC11072387

[9] Mishra AK Singh SK Dayanandan S Banerjee S Chakraborty S Gopal AB. Hypoxia-driven metabolic heterogeneity and immune evasive behaviour of gastrointestinal cancers: elements of a recipe for disaster. Cytokine. (2022) 156:155917. 10.1016/j.cyto.2022.155917 35660715

[10] Kokate SB Dixit P Das L Rath S Roy AD Poirah I. Acetylation-mediated Siah2 stabilization enhances PHD3 degradation in *Helicobacter pylori*-infected gastric epithelial cancer cells. FASEB J. (2018) 32:5378–89. 10.1096/fj.201701344RRR 29688807

[11] Rath S Das L Kokate SB Pratheek BM Chattopadhyay S Goswami C. Regulation of Noxa-mediated apoptosis in *Helicobacter pylori*-infected gastric epithelial cells. FASEB J. (2015) 29:796–806. 10.1096/fj.14-257501 25404713 PMC4422360

[12] D'Elios MM Manghetti M De Carli M Costa F Baldari CT Burroni D. T helper 1 effector cells specific for *Helicobacter pylori* in the gastric antrum of patients with peptic ulcer disease. J Immunol. (1997) 158:962–7. 10.4049/jimmunol.158.2.962 8993017

[13] Aguilar C Pauzuolis M Pompaiah M Vafadarnejad E Arampatzi P Fischer M. *Helicobacter pylori* shows tropism to gastric differentiated pit cells dependent on urea chemotaxis. Nat Commun. (2022) 13:5878. 10.1038/s41467-022-33165-4 36198679 PMC9535007

[14] Gravina AG Zagari RM De Musis C Romano L Loguercio C Romano M. *Helicobacter pylori* and extragastric diseases: a review. World J Gastroenterol. (2018) 24:3204–21. 10.3748/wjg.v24.i29.3204 30090002 PMC6079286

[15] Pandey S Follin-Arbelet B Pun CB Gautam DK Johannessen AC Petersen FC. *Helicobacter pylori* was not detected in oral squamous cell carcinomas from cohorts of Norwegian and Nepalese patients. Sci Rep. (2020) 10:8737. 10.1038/s41598-020-65694-7 32457404 PMC7250879

[16] Sekar R Murali P Junaid M. Quantification of *Helicobacter pylori* and its oncoproteins in the oral cavity: a cross-sectional study. Oral Dis. (2023) 29:1868–74. 10.1111/odi.14141 35092112

[17] Astl J Holy R Maute E Rotnagl J Kalfert D Drnkova B. Genome of *Helicobacter pylori* and serotype of HPV detected in oropharyngeal and laryngeal cancer and chronic inflammation patients. Int J Environ Res Public Health. (2021) 18:9545. 10.3390/ijerph18189545 34574466 PMC8470705

[18] Varon C Azzi-Martin L Khalid S Seeneevassen L Menard A Spuul P. Helicobacters and cancer, not only gastric cancer? Semin Cancer Biol. (2022) 86(Pt 2):1138–54. 10.1016/j.semcancer.2021.08.007 34425210

[19] Zhou D Guan WB Wang JD Zhang Y Gong W Quan ZW. comparative study of clinicopathological features between chronic cholecystitis patients with and without *Helicobacter pylori* infection in gallbladder mucosa. PLoS ONE. (2013) 8:e70265. 10.1371/journal.pone.0070265 23936177 PMC3728185

[20] Kunovsky L Dite P Jabandziev P Dolina J Vaculova J Blaho M. *Helicobacter pylori* infection and other bacteria in pancreatic cancer and autoimmune pancreatitis. World J Gastrointest Oncol. (2021) 13:835–44. 10.4251/wjgo.v13.i8.835 34457189 PMC8371525

[21] Ndawula EM Owen RJ Mihr G Borman P Hurtado A. *Helicobacter pylori* bacteraemia. Eur J Clin Microbiol Infect Dis. (1994) 13:621. 10.1007/BF01971319 7805696

[22] Alarcon T Llorca L Perez-Perez G. Impact of the microbiota and gastric disease development by *Helicobacter pylori*. Curr Top Microbiol Immunol. (2017) 400:253–75. 10.1007/978-3-319-50520-6_11 28124157

[23] Ferreira RM Pereira-Marques J Pinto-Ribeiro I Costa JL Carneiro F Machado JC. Gastric microbial community profiling reveals a dysbiotic cancer-associated microbiota. Gut. (2018) 67:226–36. 10.1136/gutjnl-2017-314205 29102920 PMC5868293

[24] Zheng W Zhu Z Ying J Long G Chen B Peng K. The effects of *Helicobacter pylori* infection on gastric microbiota in children with duodenal ulcer. Front Microbiol. (2022) 13:853184. 10.3389/fmicb.2022.853184 35547124 PMC9082302

[25] Liatsos C Papaefthymiou A Kyriakos N Galanopoulos M Doulberis M Giakoumis M. *Helicobacter pylori*, gastric microbiota and gastric cancer relationship: unrolling the tangle. World J Gastrointest Oncol. (2022) 14:959–72. 10.4251/wjgo.v14.i5.959 35646287 PMC9124990

[26] Lertpiriyapong K Whary MT Muthupalani S Lofgren JL Gamazon ER Feng Y. Gastric colonisation with a restricted commensal microbiota replicates the promotion of neoplastic lesions by diverse intestinal microbiota in the *Helicobacter pylori* INS-GAS mouse model of gastric carcinogenesis. Gut. (2014) 63:54–63. 10.1136/gutjnl-2013-305178 23812323 PMC4023484

[27] Robinson KM Crabtree J Mattick JS Anderson KE Dunning Hotopp JC. Distinguishing potential bacteria-tumor associations from contamination in a secondary data analysis of public cancer genome sequence data. Microbiome. (2017) 5:9. 10.1186/s40168-016-0224-8 28118849 PMC5264480

[28] Tsay FW Hsu PI. *H. pylori* infection and extra-gastroduodenal diseases. J Biomed Sci. (2018) 25:65. 10.1186/s12929-018-0469-6 30157866 PMC6114542

[29] Testerman TL Morris J. Beyond the stomach: an updated view of *Helicobacter pylori* pathogenesis, diagnosis, and treatment. World J Gastroenterol. (2014) 20:12781–808. 10.3748/wjg.v20.i36.12781 25278678 PMC4177463

[30] Oshima T Ozono R Yano Y Oishi Y Teragawa H Higashi Y. Association of *Helicobacter pylori* infection with systemic inflammation and endothelial dysfunction in healthy male subjects. J Am Coll Cardiol. (2005) 45:1219–22. 10.1016/j.jacc.2005.01.019 15837252

[31] Niccoli G Franceschi F Cosentino N Giupponi B De Marco G Merra G. Coronary atherosclerotic burden in patients with infection by CagA-positive strains of *Helicobacter pylori*. Coron Artery Dis. (2010) 21:217–21. 10.1097/MCA.0b013e3283399f36 20389238

[32] Choi MS Ze EY Park JY Shin TS Kim JG. *Helicobacter pylori* -derived outer membrane vesicles stimulate interleukin 8 secretion through nuclear factor kappa B activation. Korean J Intern Med. (2021) 36:854–67. 10.3904/kjim.2019.432 33242939 PMC8273812

[33] Yang S Xia YP Luo XY Chen SL Li BW Ye ZM. Exosomal CagA derived from *Helicobacter pylori*-infected gastric epithelial cells induces macrophage foam cell formation and promotes atherosclerosis. J Mol Cell Cardiol. (2019) 135:40–51. 10.1016/j.yjmcc.2019.07.011 31352044

[34] Olofsson A Vallstrom A Petzold K Tegtmeyer N Schleucher J Carlsson S. Biochemical and functional characterization of *Helicobacter pylori* vesicles. Mol Microbiol. (2010) 77:1539–55. 10.1111/j.1365-2958.2010.07307.x 20659286 PMC3068288

[35] Brusselaers N Wahlin K Engstrand L Lagergren J. Maintenance therapy with proton pump inhibitors and risk of gastric cancer: a nationwide population-based cohort study in Sweden. BMJ Open. (2017) 7:e017739. 10.1136/bmjopen-2017-017739 29084798 PMC5665226

[36] Coker OO Dai Z Nie Y Zhao G Cao L Nakatsu G. Mucosal microbiome dysbiosis in gastric carcinogenesis. Gut. (2018) 67:1024–32. 10.1136/gutjnl-2017-314281 28765474 PMC5969346

[37] Liu Z Zhang D Chen S. Unveiling the gastric microbiota: implications for gastric carcinogenesis, immune responses, and clinical prospects. J Exp Clin Cancer Res. (2024) 43:118. 10.1186/s13046-024-03034-7 38641815 PMC11027554

[38] Zahmatkesh ME Jahanbakhsh M Hoseini N Shegefti S Peymani A Dabin H. Effects of exosomes derived from *Helicobacter pylori* outer membrane vesicle-infected hepatocytes on hepatic stellate cell activation and liver fibrosis induction. Front Cell Infect Microbiol. (2022) 12:857570. 10.3389/fcimb.2022.857570 35832384 PMC9271900

[39] Carlsohn E Nystrom J Karlsson H Svennerholm AM Nilsson CL. Characterization of the outer membrane protein profile from disease-related *Helicobacter pylori* isolates by subcellular fractionation and nano-LC FT-ICR MS analysis. J Proteome Res. (2006) 5:3197–204. 10.1021/pr060181p 17081072

[40] Guo Y Xu C Gong R Hu T Zhang X Xie X. Exosomal CagA from *Helicobacter pylori* aggravates intestinal epithelium barrier dysfunction in chronic colitis by facilitating Claudin-2 expression. Gut Pathog. (2022) 14:13. 10.1186/s13099-022-00486-0 35331316 PMC8944046

[41] Whitworth DE Morgan BH. Synergism Between Bacterial GAPDH and OMVs: disparate mechanisms but co-operative action. Front Microbiol. (2015) 6:1231. 10.3389/fmicb.2015.01231 26617577 PMC4637417

[42] Zwarycz AS Page T Nikolova G Radford EJ Whitworth DE. Predatory strategies of myxococcus xanthus: prey susceptibility to OMVs and moonlighting enzymes. Microorganisms. (2023) 11:874. 10.3390/microorganisms11040874 37110297 PMC10141889

[43] Liu Y Defourny KAY Smid EJ Abee T. Gram-positive bacterial extracellular vesicles and their impact on health and disease. Front Microbiol. (2018) 9:1502. 10.3389/fmicb.2018.01502 30038605 PMC6046439

[44] Qing S Lyu C Zhu L Pan C Wang S Li F. Biomineralized bacterial outer membrane vesicles potentiate safe and efficient tumor microenvironment reprogramming for anticancer therapy. Adv Mater. (2020) 32:e2002085. 10.1002/adma.202002085 33015871

[45] Morandi MI Busko P Ozer-Partuk E Khan S Zarfati G Elbaz-Alon Y. Extracellular vesicle fusion visualized by cryo-electron microscopy. PNAS Nexus. (2022) 1:pgac156. 10.1093/pnasnexus/pgac156 36714848 PMC9802263

[46] Gnopo YMD Misra A Hsu HL DeLisa MP Daniel S Putnam D. Induced fusion and aggregation of bacterial outer membrane vesicles: experimental and theoretical analysis. J Colloid Interface Sci. (2020) 578:522–32. 10.1016/j.jcis.2020.04.068 32540551 PMC7487024

[47] Karmacharya M Kumar S Cho YK. Tuning the extracellular vesicles membrane through fusion for biomedical applications. J Funct Biomater. (2023) 14:117. 10.3390/jfb14020117 36826916 PMC9960107

[48] Yang Y Hong Y Nam GH Chung JH Koh E Kim IS. Virus-mimetic fusogenic exosomes for direct delivery of integral membrane proteins to target cell membranes. Adv Mater. (2017) 29. 10.1002/adma.201605604 28165174

[49] Du S Guan Y Xie A Yan Z Gao S Li W. Extracellular vesicles: a rising star for therapeutics and drug delivery. J Nanobiotechnology. (2023) 21:231. 10.1186/s12951-023-01973-5 37475025 PMC10360328

